# An Economic Evaluation of Direct Oral Penicillin Challenge for De‐Labelling Low Risk Patients With a Penicillin Allergy Label

**DOI:** 10.1111/cea.14633

**Published:** 2025-02-05

**Authors:** R. Bestwick, R. Bhogal, K. Kildonaviciute, B. Y. Ng, B. Jackson, C. Moriarty, C. Thomas, L. Savic, S. A. Misbah, M. T. Krishna, R. Mujica‐Mota

**Affiliations:** ^1^ Academic Unit of Health Economics Leeds Institute of Health Sciences, University of Leeds Leeds UK; ^2^ Department of Pharmacy University Hospitals Birmingham NHS Foundation Trust Birmingham UK; ^3^ Department of Pharmacy Oxford University Hospitals NHS Foundation Trust Oxford UK; ^4^ Theatres and Anaesthetics Research Team St James' University Hospital, Leeds Teaching Hospitals Leeds UK; ^5^ Department of Anaesthesia St James' University Hospital, Leeds Teaching Hospitals NHS Trust Leeds UK; ^6^ Department of Clinical Immunology Oxford University Hospitals NHS Foundation Trust Oxford UK; ^7^ Institute of Immunology and Immunotherapy, University of Birmingham Birmingham UK; ^8^ Department of Allergy and Immunology University Hospitals Birmingham NHS Foundation Trust Birmingham UK

**Keywords:** cost analysis, direct oral penicillin challenge, health economics, low risk, penicillin allergy, risk stratification

## Abstract

**Background:**

Removing inaccurate penicillin allergy labels (PALs) can reduce unnecessary exposure to ‘watch’ and ‘reserve’ groups of antibiotics and thereby reduce antimicrobial resistance. The most efficient model for a non‐allergy‐specialist‐led penicillin allergy de‐labelling (PADL) service has not been established.

**Objective:**

To determine the costs to the UK National Health Service of a direct oral penicillin challenge (DPC) for low‐risk patients with a PAL in three hospitals in England, each with a different non‐allergy‐specialist delivery model: pharmacist‐led, nurse‐led, and mixed multidisciplinary.

**Methods:**

Cost analysis of the DPC pathway, including resources related to staff time and antibiotics. The effect of de‐labelling on healthcare utilisation over 5 years was modelled using data from the published literature.

**Results:**

In total, 2257 patients from the Acute Medical or Infectious Disease Unit (AMU/IDU), Pre‐surgical, and Haematology‐Oncology departments were screened. Subsequently, 126 underwent DPC, and 122 were de‐labelled. Twenty‐two of these were de‐labelled in time to affect their antibiotic regimen; 6 from AMU/IDU and 16 Pre‐surgery. The DPC represented 22%–23% of the pathway cost in the pharmacist‐led and mixed models, and 15% in the nurse‐led model. Across departments and models, the cost per de‐labelled patient varied between £577 (95% Credible Interval: 370, 633) for haematology‐oncology patients to £2329 (947, 19,504) for AMU/IDU patients, both under the nurse‐led model. After 5 years, recouping costs was unlikely for AMU/IDU patients under any model or for all patients combined under the mixed model.

**Conclusions:**

The penicillin allergy de‐labelling pathway cost was  ≥ 4‐fold that of the DPC alone. Costs were up to 3 times higher in an acute compared to an elective setting. No short‐term cost savings were identified from proactive or opportunistic penicillin allergy de‐labelling in this study.

## Graphical abstract

The cost of the Direct Oral Penicillin Challenge (DPC) itself is only a fraction of the full costs required to test for a penicillin allergy label by non‐allergists. The costs of DPC varied between hospitals using a nurse‐led, pharmacist‐led, or mixture of pharmacists and nurses. In the mixed model, the cost of therapeutic delabelling was more than three times that for delabelling elective patients. The most promising model of care is nurse‐led, and the pharmacist‐led is the most fruitful area of further research.

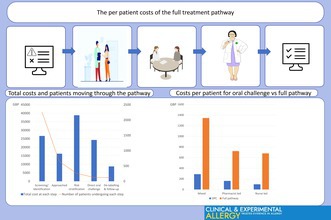

**Summary**

- We have evaluated the health economic impact of Penicillin Allergy Delabelling through Oral Challenges.
- Penicillin challenges accoungt for less than 25% of the total costs of a delabelling pathway.
- Delabelling had negligible short‐term cost savings and is uncertain to recoup costs over 5 years.

## 1 Introduction

Around 90% of hospitalised patients with a penicillin allergy label (PAL) do not have a true allergy [1]. The current pathway to de‐label incorrect PALs in most of the world involves taking a medical history, directly de‐labelling those with a history incompatible with a penicillin allergy, and in the majority of the remaining individuals, specifically those with lower risk histories, proceeding with a supervised oral penicillin challenge to confirm current tolerance. Penicillin skin testing, typically with penicilloyl‐polylisine, is now reserved for individuals considered to be at high risk of an IgE‐mediated allergy prior to a confirmatory oral challenge, if skin test negative, because there is a high false positive rate for penicillin skin testing [2, 3, 4, 5, 6]. Patients with a PAL describing symptoms suggestive of a non‐immune‐mediated adverse reaction, benign rash, or those that acquired a label due to family history without an index reaction are deemed low‐risk [7]. For this group, a direct oral penicillin challenge (DPC; without undertaking skin tests) has been reported to be a safe, feasible, and simpler alternative to skin testing [8, 9, 10]. There is emerging evidence that DPC can be delivered by non‐allergy specialist healthcare professionals (HCPs) [11, 12, 13], and the BSACI has released supportive clinical guidance regarding the set‐up of non‐allergist‐led de‐labelling services in secondary care [7]. The WHO AWaRe framework emphasises the importance of prescribing ‘Access’ groups (e.g., penicillins) as first‐line antibiotics for common infections and avoiding unnecessary use of higher‐class antibiotics (Watch and Reserve) as a global strategy to reduce rates of antimicrobial resistance [14].

Previous studies conducted in the US and Australia have shown that the cost of DPC in low‐risk patients may be offset by savings in the cost of antibiotic use within future infection‐related episodes [15, 16]. However, these studies only determined the cost of DPC per se rather than the full DPC pathway (which additionally includes the activities required to identify and risk stratify patients). Moreover, previous studies have not investigated the variation in DPC costs between elective and acute clinical settings and pharmacist versus nurse non‐allergy‐specialist‐led DPC models.

The objective of this study was to evaluate the costs and potential cost‐effectiveness of DPC in low‐risk patients with a PAL, relative to standard care, from the perspective of the UK NHS and personal social services, which in our study settings are covered by a tax‐funded system.

## 2 Materials and Methods

Since our study followed up patients until 5 days after testing and no adverse events occurred within that period, no differences in clinical outcomes were observed. We therefore conducted a cost analysis using data collected from three centres: University Hospitals Birmingham NHS Foundation Trust, Oxford University Hospitals NHS Foundation Trust, and Leeds Teaching Hospitals NHS Trust. All study personnel attended pre‐study workshops for training and standardisation of procedures.

The DPC model differed between sites by the professional background of study personnel: research nurse (RN) led at Leeds, research pharmacist (RP) led at Birmingham (with RN input for screening), and RP led at Oxford.

This study was approved by The London Bridge Ethics Committee (REC Reference 21/PR/0814; IRAS project ID: 293544).

### 2.1 Mapping of Treatment Pathways

To aid mapping of the DPC intervention and current usual care pathways for patients with a PAL, interviews were held with HCPs from the study sites. These took place between March and June 2022 and involved three RPs (one at Birmingham, two at Oxford), two RNs (Leeds), and six consultants (one oncologist, three surgeons, and two infectious disease/microbiologists). We also discussed our methods and early results with members of the Patient and Public Involvement Advisory Group.

### 2.2 Development of DPC Pathway Model

The interviews revealed that the three sites have access to regional specialist outpatient‐based drug allergy services and not routine urgent inpatient de‐labelling services.

**FIGURE 1:**
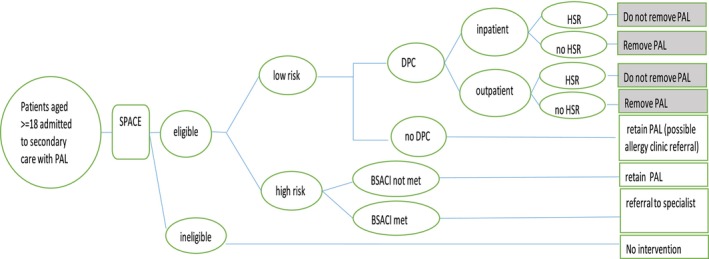
Decision Tree for DPC. BSACI, British Society for Allergy and Clinical Immunology criteria; DPC, direct oral penicillin challenge; HSR, hypersensitivity reaction; PAL, penicillin allergy label; SPACE, Start of new DPC pathway investigated in an observational multicentre feasibility study of direct oral penicillin challenge in de‐labelling ‘low‐risk’ patients with penicillin allergy by non‐allergy healthcare professionals (SPACE study). Circles represent chance events in the DPC pathway moving from identifying an adult patient with PAL, on the far left of the pathway diagram, through assessment of eligibility, risk stratification, and, for patients deemed low risk, the probability of undergoing DPC or not undergoing DPC as inpatient or outpatient with associated outcomes, on the far right, of de‐labelled (‘PAL removal’) or not de‐labelled (Do not remove PAL’). Shaded boxes reflect outcomes that would be altered by DPC relative to outcomes that would have been observed under the counterfactual standard of care pathway.

Therefore, the comparator in our analysis was ‘no testing and manage according to the PAL’, and we assumed that any potential effect of DPC was likely to be limited to low‐risk patients successfully de‐labelled. Figure 1 depicts the DPC pathway as implemented during our observational study (SPACE study), which investigated the feasibility of DPC in de‐labelling ‘low‐risk’ patients with penicillin allergy by non‐allergy healthcare professionals.

In Figure 1, admitted adult patients are identified from the Acute Medical or Infectious Diseases Unit (AMU/IDU), pre‐surgical unit, or haematology‐oncology. They are screened for potential eligibility (using predetermined criteria) and then approached for expression of interest (Table S1). Informed consent was obtained for those willing to participate, and risk stratification was conducted as per study criteria (Supporting Information). Some eligible patients were unreachable, not approached due to logistical challenges during the pandemic (restrictions to access patients on COVID wards due to infection risks, rapid turnover, patient discharge or transfer prior to the research team being able to make contact), or approached but declined to participate. The proportion of those who were approached out of those deemed eligible during the screening process is hereafter referred to as the ‘coverage rate’.

$$\begin{array}{c} \text{coverage rate}=\frac{\text{patients approached for risk stratficiation}}{\text{Eligible patients}\;\left(\text{identified during screening process}\right)} \end{array}$$

After risk stratification, ‘high‐risk’ patients meeting the BSACI referral criteria [17] had an outcome letter sent to their general practitioner (GP) recommending onwards referral to an allergy specialist. Patients deemed ‘low‐risk’ were offered a DPC. This study offered 2 types of de‐labelling. ‘Opportunistic de‐labelling’ involved administration of DPC at a time when the patient did not require antibiotics, i.e., intervention was offered primarily for de‐labelling. A 500 mg amoxicillin oral tablet was given, followed by 250 mg amoxicillin twice daily for 3 days. There are no validated protocols regarding the dose and duration of DPC. The protocol for DPC employed in this study is based on consensus within the research team at the time of funding acquisition and was adapted from specialist clinical practice [18, 19]. ‘Therapeutic de‐labelling’ involved exclusion of type‐1 hypersensitivity with 500 mg of amoxicillin followed by a full therapeutic course of amoxicillin or a penicillin‐containing antibiotic based on clinical indication for treatment of infection.

Those who underwent DPC were followed up to day 5 to assess for a delayed/nonimmediate hypersensitivity reaction. On day 5, for inpatients who were still on the ward, a direct clinical review was undertaken; no patient was kept in hospital because of the 5‐day review. Those discharged or who underwent DPC in an elective setting were contacted via telephone. Any emergent or severe adverse events were fully evaluated by the study team, and if deemed unrelated to DPC, the patient was de‐labelled; otherwise, the PAL was retained in records.

**FIGURE 2:**
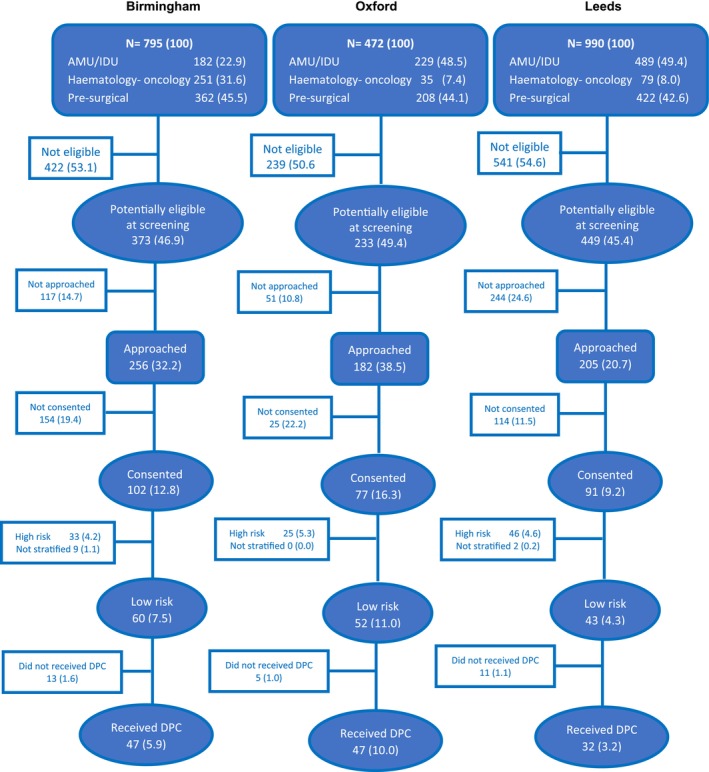
Characteristics and flow of patients in the three study sites. DPC: Direct Penicillin Challenge. AMU, Acute Medical Unit; IDU, Infectious Diseases Unit. Risk stratification: 259 out of 270 (96%) consented patients were risk stratified: 11 could not undergo the stratification process due to practical reasons such as change in their circumstances and not being able to reach them despite multiple attempts.

Figure 2 presents the characteristics of screened patients with a PAL. Across sites, the proportion of eligible patients was similar; however, fewer could be approached in Leeds (lower coverage rate), where AMUs were spread across multiple wards during the pandemic, and it was therefore more difficult for research staff to contact clinical teams and patients. The cohorts also differed by patient age: 26.5% of those recruited at Leeds were over 80 years old, compared with 13.8% from Birmingham and 24.4% in Oxford.

### 2.3 Data Collection and Sources

Data collected prospectively included demographics, time inputs and outcomes of screening and risk stratification, and DPC outcome. The DPC step was standardised across all centres, and time input/duration for this was collected retrospectively using a questionnaire completed by respective study HCPs. Other retrospectively collected data were initial and revised antibiotic medication regimens among de‐labelled patients in AMU/IDU and local Trust‐recommended antibiotic regimens for the surgery undergone by de‐labelled pre‐surgical patients. Observing revised antibiotic medication in haematology‐oncology and pre‐surgical patients was beyond the scope of our 5‐day follow‐up study end point.

### 2.4 Intervention Costs

The costs of delivering the DPC arose from staff time and medications. We used a matrix to map individual staff inputs (collected from the questionnaire) to the different steps in the DPC pathway, and staff time was evaluated using routinely published unit costs (considering HCP role and Agenda for Change (AfC) band) to include salary, on‐costs, management and non‐care staff, non‐staff overheads, and space use [20]. We accounted for any time that study consultants directly input into DPC consultation, as recorded retrospectively by the study HPC at each site.

The cost of oral amoxicillin was obtained from the electronic market information tool (eMIT) [21]. No patient in the study had an anaphylactic episode, and therefore the costs of such episodes were not included. The cost of monitoring equipment, such as thermometers, was negligible and not measured. Unit costs of resource inputs are shown in Table S2.

We also estimated the costs of staff delivering and attending the study training workshops in scenario analysis, where we assumed that the skills lasted 2 years and applied a 5% annual discount rate.

We used prospectively collected electronic individual patient data [22, 23] on the screening and risk stratification steps to incorporate uncertainty in retrospective cost estimates (Table 1, Table S3).

**TABLE 1:** Parameters for extrapolation of costs of DPC.

Parameter	Point estimate	Distribution	Central tendency and dispersion parameters	Notes
**Prevalence**				
Eligibility	0.47	Beta	Alpha: 1055 Beta: 1202	Value in the whole SPACE study cohort; analyses were based on subgroup‐specific values
Approach rate (coverage)	0.61	Beta	Alpha: 643 Beta: 412	Out of those eligible in the whole SPACE study cohort; analyses were based on subgroup‐specific values
Consent & Risk stratified	0.42	Beta	Alpha: 270 Beta: 373	Out of those approached in the whole SPACE study cohort; analyses were based on subgroup‐specific values
Low risk & Undergoes DPC	0.47	Beta	Alpha: 126 Beta: 144	Out of those consented and risk stratified in the whole SPACE study cohort, analyses were based on subgroup‐specific values
De‐labelled	0.97	Beta	Alpha: 122 Beta: 4	Out of those DPC tested in the whole SPACE study cohort, analyses were based on subgroup‐specific values
**Resource use quantities**				
Mean annual number of GP contacts avoided per de‐labelled patient over 4.5 years	3.052	Normal	Mean: 3.052 SE: 0.375	Mean difference in annual number of GP contacts between primary care patients with a PAL and patients without a PAL matched for age, gender, and follow‐up period from electronic medical records in Utrecht over a median 4.5 follow‐up [24]
12‐week re‐admission rate: de‐labelled patients	0.16	Fixed	N/A	The percentage of emergency readmissions to any hospital in England occurring within 30 days of the most recent discharge from hospital, HES (reporting period 01/04/2021 to 31/03/2022), extrapolated to 90‐day readmission using the ratio of 12‐ to 4‐week readmission rate reported by van Dijk et al. [25, 26]
Relative Risk Ratio of 12‐week readmissions: PAL versus de‐labelled	1.28	Log normal	Mean: 0.25 SE: 0.08	12‐week relative risk ratio of hospital readmission of patients with PAL versus without PAL [26]
Mean LOS days avoided by de‐labelling vs. PAL (index admission & readmissions)	0.19	Fixed	N/A	Calculated based on data from Table VI in Powell et al. [27]
**Unit costs**				
Screening	8–18	Normal	Mean: 8–18 SE: 0.17–0.37	Range of mean unit cost across the three study sites; SE derived from individual patient REDCap data at Birmingham & Oxford sites (Table S3). As Leeds did not collect sufficient data, its SE was imputed using the coefficient of variation in the respective Birmingham and Oxford data
Approach	10–33	Fixed	N/A	Range of mean unit cost across the three study sites
Risk stratification	52–217	Normal	Mean: 52–217 SE: 0.51–1.13	Range of mean unit cost across the three study sites; SE derived from individual patient REDCap data at Birmingham & Oxford sites (Table S3). As Leeds did not collect sufficient data, its SE was imputed using the coefficient of variation in the respective Birmingham and Oxford data
DPC	84–282	Fixed	N/A	Range of mean unit cost across the three study sites; cost of medications was £0.03–0.17 [21]
De‐labelling	13–92	Fixed	N/A	Range of mean unit cost across the three study sites
Cost of hospital (re)admission	2634	Fixed	N/A	Weighted average for the non‐elective admission categories from the National Schedule of NHS Costs financial year 2020–21 [28]
Cost per excess bed day	286	Fixed	NA	Average of HRG cost for APC days exceeding trim point for each HRG, 2019 National Tariff [29]
Cost per GP contact	28	Fixed	NA	Cost per GP visit, Jones and Burns [20]
Prescription costs	31	Fixed	NA	Prescription costs per GP visit: Jones and Burns [20]

Abbreviations: LOS, Length of hospital stay; PAL, Penicillin Allergy Label; SE, Standard error.

### 2.5 Costs of Antibiotic Medication Use at Index Hospital Admission

Cost savings associated with antibiotic use in ‘therapeutically de‐labelled’ patients were determined by the cost difference between the antibiotic regimen the patients were anticipated to receive at time of screening and the regimen they were switched to following de‐labelling. Costs included drug acquisition and excluded administration and preparation.

Cost differences in de‐labelled pre‐surgical patients were inferred from the respective hospital guidelines for the peri‐procedure antibiotic prophylaxis of the surgery they were listed for, with and without PAL.

### 2.6 Extrapolation

As part of a separate unpublished study, we systematically searched the literature to identify DPC economic modelling studies with analytical time horizons beyond index DPC discharge. The search was last updated in full on 14 November 2023 (Supporting Information). We use data from these identified studies to assess the impact of DPC beyond the end of follow‐up in our study [30].

We evaluated cost impacts on length of stay (LOS) in initial admission, occurrence and LOS in 90 day readmissions, and GP contacts and associated prescriptions over a 4.5‐year time horizon using relevant recent published UK cost and European outcome data [24, 25, 26, 27, 28, 29]. Costs after the first year were discounted at an annual rate of 3.5%.

Table 1 presents the parameter values for the different resource use related to clinical events in our extrapolation. Details are found in the Supporting Information.

### 2.7 Sensitivity Analyses: Intervention Costs

We investigated the variation of pathway costs as a function of the coverage rate and the following assumptions:

- Administrative & data manager support were available at Oxford
- Optimisation of the Birmingham model by AfC Band 6/7 RNs undertaking the identification and screening instead of the senior RP (AfC Band 8a)
- Screening and day 5 follow‐up incur zero additional costs on existing services

### 2.8 Statistical and Probabilistic Analysis

Results of DPC pathway costs are presented alongside their associated 95% Credible Intervals (CrI) based on 1000 random samples of the probability of patients progressing through the DPC steps from identification through to risk stratification and DPC and being de‐labelled.

### 2.9 Value of Information Analysis

We calculated the expected value of conducting a longitudinal study to investigate the net benefit (overall cost savings to the NHS net of DPC costs) of the different delivery models in our study patient cohort over 4.5 years [30, 31].

Costs were expressed in GBP at prices of 2021. Results are presented by study site and clinical setting; variation across other characteristics was not analysed. Details of the analysis plan developed in collaboration with clinical lead investigators are available from the authors. The funder of the study (National Institute for Health and Care Research) had no role in study design, data collection, data analysis, data interpretation, or writing of the report.

## 3 Results

### 3.1 Costs of the DPC

**FIGURE 3:**
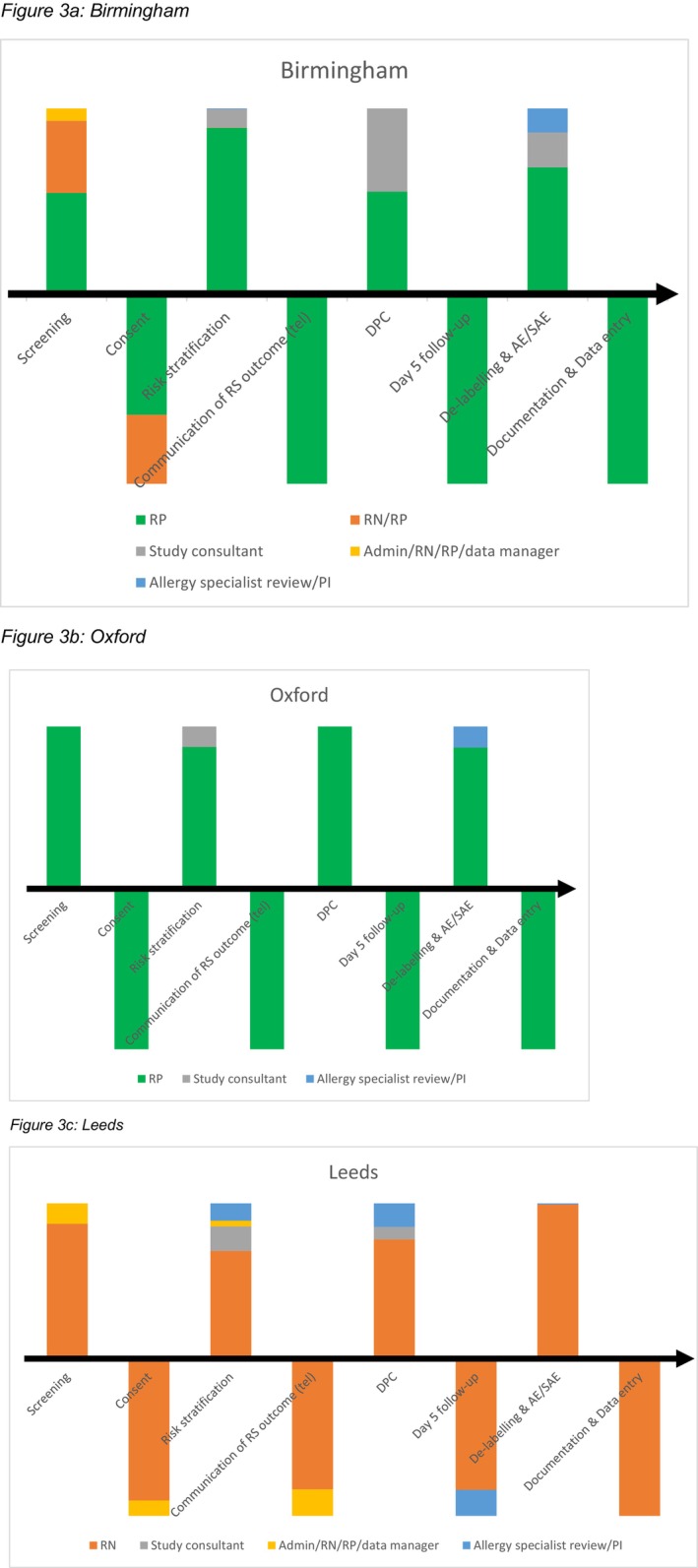
DPC models in the three study sites by costed staff inputs. (a) Birmingham. (b) Oxford. (c) Leeds. AE, adverse event; PI, principal investigator; RN, research nurse; RP, research pharmacist; SAE, severe adverse event. Risk stratification.

The DPC model varied across the three sites with respect to staff mix and time inputs at each pathway step. Figure S1 and Figure 3 illustrate the cost of each step and the relative contribution of different staff categories. Detailed results of patient characteristics are published elsewhere [23].

### 3.2 Birmingham

Costs of staff time delivering DPC in this cohort were £68,263, which included £47,187 and £9029 for RP and RN time, respectively. Screening activities accounted for 24% of total non‐training costs (Table S4). Risk stratification and DPC, respectively, constituted 32% and 22% of non‐training costs. Whilst the oral challenge itself cost £288 per patient tested, the overall costs per tested patient increased to £1209 when accounting for staff time inputs for the other pathway activities (Table 2).

**TABLE 2:** Deterministic staff costs of SPACE intervention at the three study sites (£).

	Total cost at each step (a)	Number of patients undergoing each step (b)	Cost per patient at each step (a/b)	Total cumulative costs at each step (c)	Total cumulative cost per patient at each step (c/b)
**BIRMINGHAM**					
Screening/Identification	14,281	795	18	14,281	18
Approached	8426	256	33	22,707	89
Risk stratification^a^	20,608	93	222	43,315	466
Direct oral challenge	13,528	47	288	56,843	1209
De‐labelling & follow‐up	3626	45	81	60,469	1344
**OXFORD**					
Screening/Identification	4434	472	9	4434	9
Approached	3279	182	18	7713	42
Risk stratification^a^	13,462	77	175	21,175	275
Direct oral challenge	7623	47	162	28,798	613
De‐labelling & follow‐up	4423	46	96	33,221	722
**LEEDS**					
Screening/Identification	7947	990	8	7963	8
Consent	4521	205	22	12,483	61
Risk stratification^a^	4739	89	53	17,222	194
Direct oral challenge	3144	32	98	20,366	636
De‐labelling & follow‐up	698	31	23	21,064	679

^a^ Includes ‘Communication of risk stratification outcome via telephone’.

### 3.3 Oxford

Costs of staff time delivering DPC in this cohort were £36,565, including £32,569 for RPs (who performed the data management themselves). Risk stratification activities accounted for the largest share of total non‐training costs (39%) (Table S5). Screening combined with consent amounted to 23%, and oral challenge constituted 23% of non‐training costs. The oral challenge itself cost £162 per patient tested, and costs increased to £613 per patient when accounting for staff time costs of the overall pathway (Table 2).

### 3.4 Leeds

Costs of staff time delivering DPC in this cohort were £24,691, including £19,301 of RN time. Screening accounted for the largest share of total non‐training costs, 38% (Table S6). Risk stratification and oral challenge itself represented 22% and 15% of non‐training costs, respectively. The oral challenge cost £98 per patient tested, and the costs of staff time inputs for the full pathway were £636 per tested patient (Table 2).

### 3.5 Costs of DPC Net of Savings in Antibiotic Medication Use

#### 3.5.1 Presurgical Patients

Across study sites, 26 patients were de‐labelled before their surgery. Sixteen of these were listed for procedures that recommended antibiotic prophylaxis. Of these, eight patients had an antibiotic recommendation that differed by PAL status. The mean difference between the cost of antibiotic therapy recommended for their operations with and without PAL (according to the local hospital guideline) was −£1.95, or −£0.44 per de‐labelled patient (Table S7).

#### 3.5.2 Therapeutically De‐Labelled Patients (AMU/IDU)

Data was analysed for six patients who underwent therapeutic de‐labelling (the remaining 12 were opportunistically delabelled patients and thus incurred zero cost savings) in AMU. Where PAL was removed, the average antibiotic cost per treatment course was £1.49 less than if they had retained the PAL (Table S8). Overall, the AMU cost saving corresponds to a £0.07 saving per de‐labelled patient.

Overall, antibiotic cost savings reduced costs by 0.04% (Table S9).

#### 3.5.3 Sensitivity Analysis

**FIGURE 4:**
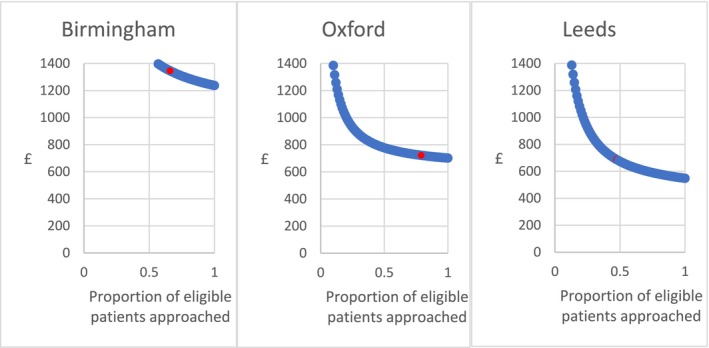
Cost per de‐labelled patient as a function of proportion of eligible patients approached at each study site. Red dot's placement represents the proportion of eligible patients approached per site in the SPACE study, and the respective cost per de‐labelled patient (estimated through probabilistic sensitivity analysis).

Figure 4 illustrates the relationship between the coverage rate and the cost per de‐labelled patient. As more patients are approached for participation, the technical efficiency of DPC increases (i.e., the cost per de‐labelled participant diminishes). There is greater scope for economies of scale in Leeds compared to the other study sites. For instance, a 10% increase in coverage rate (from 46% to 51%) at Leeds would reduce the cost per de‐labelled patient by 3.8% (from £695 to £669), whilst at Birmingham (69%–76%) it would do so by 2.1% (£1332 to £1304) and at Oxford (from 78% to 86%) by 1.2% (from £722 to £714).

Small variation in costs resulted from exploring alternative staff skill mix scenarios or including the costs of training. However, when assuming zero opportunity costs of screening and day 5 follow‐up, the pathway at Birmingham is 26% less costly at £1001 per de‐labelled patient, and 16% and 39% in Oxford (£607) and Leeds (£414), respectively (Tables S10 and S11).

### 3.6 Extrapolated Net Costs of DPC

**FIGURE 5:**
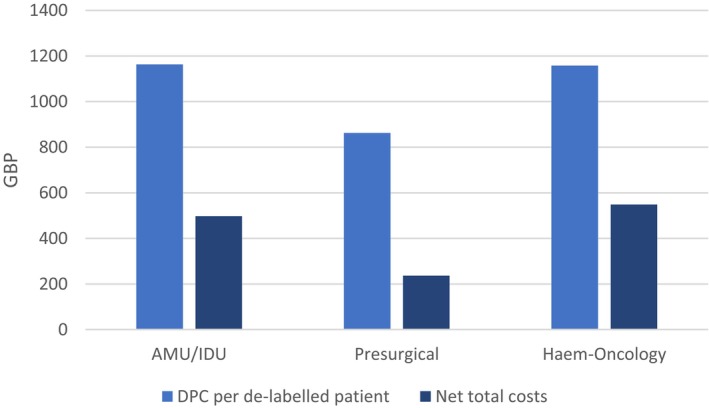
Cost per de‐labelled patient and net DPC costs by clinical settings.

Overall, the cost per de‐labelled patient in AMU/IDU was £1163, and the respective cost for patients in elective settings was £908 (Figure 5), with the former setting being up to 4 times as large as the latter in the Leeds site (Table 3). In most patient groups, the observed costs of DPC per de‐labelled patient are larger than the extrapolated cost savings over 4.5 years after DPC (Table 3). The difference in the costs of DPC and cost savings (total net costs) per patient ranges from −£108 for haematology‐oncology patients in Leeds to £1663 for AMU/IDU in Leeds.

**TABLE 3:** Extrapolated costs by patient subgroup and study site costs in GBP (95% CI).

	AMU/IDU	Pre‐surgical patients	Haematology‐oncology
**Discounted cost impact per de‐labelled patient over 4.5 years**			
Change in LOS initial spell^a^, ^b^, ^c^	−18	−5	0
Change in occurrence and LOS of 90‐day readmissions^c^	−46	−18	−7
Change in GP contacts at 4.5 years (discounted)^d^	−287	−287	−287
Change in GP prescription costs at 4.5 years (discounted)^d^	−316	−316	−316
Total (A)	−667	−626	−610
**Birmingham**			
DPC per de‐labelled patient (B)	1829 (1115, 4943)	1280 (1027, 1608)	1483 (1085, 2206)
Net total costs (B‐A)	1163 (426, 4262)	655 (346, 997)	874 (442, 1558)
Probability of DPC being cost‐saving (i.e., A > C) %	0	0	0
**Oxford**			
DPC per de‐labelled patient (C)	747 (627, 1041)	715 (582, 811)	707 (520, 1207)
Net total costs (C‐A)	81 (−129, 390)	89 (−145, 222)	98 (−149, 619)
Probability of DPC being cost‐saving (i.e., A > C) %	21	20	21
**Leeds**			
DPC per de‐labelled patient (D)	2329 (947, 19,504)	577 (370, 633)	502 (269, 1438)
Net total costs (D‐A)	1663 (262, 18,832)	−49 (−350, 43)	−108 (−405, 823)
Probability of DPC being cost‐saving (i.e., A > C) %	0	68	58

^a^ By pre‐surgical patients de‐labelled before surgery.

^b^ For therapeutically de‐labelled patients.

^c^ Based on estimated LOS differences between spells of adult patients admitted to a medium‐sized hospital in Truro over a 2‐year period with and without a penicillin allergy record for whom an antibiotic was used [27].

^d^ Based on a retrospective cohort study of primary care patients with a penicillin allergy record in the Utrecht area, the Netherlands, matched for age, gender, and follow‐up period with three patients without a PAL and followed up over 4.5 years; figures are discounted at an annual rate of 3.5% [26].

#### 3.6.1 Value of Information Analysis

The greatest value of future research on net‐zero‐cost services is expected to arise from investigating the Leeds (nurse‐led) model in haematology‐oncology patients. Overall, however, further research of the Oxford (pharmacist‐led) model presents the greater value of the three models, with little difference between which of the three patient groups is the focus of a future study (Table S12).

## 4 Discussion

We conducted an early economic evaluation for a non‐allergy‐specialist led DPC in secondary care. This involved assessing three different models of service delivery across three different clinical settings within a research framework of opportunistic and therapeutic de‐labelling. We found that performing DPCs in AMU/IDU patients cost more compared to DPC in elective settings. This is more starkly illustrated in Leeds, where the cost per de‐labelled AMU/IDU participant was 12‐fold compared to a pre‐surgical participant. This magnitude reflects the lower coverage rate among AMU/IDU patients at Leeds, which subsequently contributed to the termination of screening in this clinical setting after the first 3 months of the study; recruitment instead focused on elective settings for the remaining period.

Our data showed that the Leeds model (involving RNs) resulted in lower costs compared to Oxford and Birmingham (both RP models, although Birmingham partly employed non‐pharmacist clinical and support staff to conduct screening and administrative tasks). While the services in Oxford and Birmingham achieved close to optimal levels of technical efficiency at high coverage rates, DPC in Leeds had the potential to increase its 46% coverage rate of eligible patients to exploit economies of scale. However, our projected economies of scale relied on the assumption of constant marginal costs of increasing nurse capacity and need confirmation in a future larger study. In this regard, discussions held with local managers highlighted that additional nursing staff may be needed to enhance the timely throughput of low‐risk patients undergoing DPC so that they can benefit from pre‐surgical penicillin prophylaxis, e.g., for cardiac surgery, hepato‐biliary surgery, and arthroplasty (AntimicrobialPrescribingGuidelines.pdf (uhb.nhs.uk), accessed 17/10/24; at the time of the study, cefazolin was not used widely at the study sites).

A limitation of our analysis is that the recorded healthcare resource use involved in delivering DPC was driven by the research study protocol and therefore may overestimate costs in routine clinical practice. Several tasks involving identifying and screening patients with a PAL were conducted by senior pharmacists in Oxford and Birmingham but could perhaps have been undertaken by less experienced, but trained, clinical and non‐clinical staff. Moreover, some tasks may become standardised, embedded within routine clinical activity, and streamlined via the adoption of computerised decision support systems (CDSS), thereby reducing screening and overall costs.

Studies in other countries reported that DPC in an inpatient setting may produce enough cost savings to pay for itself [10, 15, 16]. An oral challenge in Australia cost £23 and was associated with savings in low‐risk patients of £4511 [15]. In that study, antibiotics and intravenous consumables contributed only £8 and £34, the remaining were savings from reduced LOS. Comparatively, in our small cohort of therapeutically de‐labelled patients, per‐patient cost savings were only £1.49 from antibiotic use and £53 from LOS, while the oral challenge itself cost between £98–288 depending on the centre.

In other studies, a 3‐day amoxicillin DPC conducted by an allergist cost £137 (including amoxicillin, pharmacist preparation time, and consultation cost) and was associated with direct antibiotic savings of £120 [16], and a separate case series (with matched controls) reported antibiotic savings of £170 [10]. Our results suggest that these studies underestimate the costs of delivering DPC in an inpatient setting since they omitted the costs associated with patient identification or risk stratification activities, which in our study cost an additional £257 to £963 per de‐labelled patient.

While our economic analysis is necessarily confined to the short‐term impact of PADL, we assessed potential healthcare cost savings over 4.5 years after DPC. In this scenario, the Leeds model in the haematology‐oncology cohort results in overall cost savings and therefore is likely to be attractive to the NHS. Moreover, there are long‐term benefits of appropriate de‐labelling to patients from avoiding delayed treatment of serious infections such as sepsis, meningococcal meningitis, and gas gangrene [32, 33, 34].

Our analysis of the European evidence [24, 25, 26, 27] suggests that primary care visits, hospital visits, and hospitalisation rates are important outcomes for assessing the cost‐effectiveness of PADL pathways. In a retrospective US study, 308 penicillin allergy‐tested patients were found to have 0.09 fewer outpatient visits, 0.13 fewer emergency department visits, and 0.55 fewer hospital days per year over a 3.6 year follow‐up, compared to their controls [35]. These effects would be worth £650 per patient in discounted savings; a similar impact to what we found. Whilst PALs may contribute to antimicrobial resistance [36], the health and economic impact of PADL services through this association has not yet been investigated.

Skin tests are twice as costly as DPC [37], and allergy specialists are in huge demand within the NHS [38]. DPCs circumvent the need for skin testing in low‐risk patients, and we demonstrate how a non‐allergy‐specialist‐led DPC may facilitate superior antimicrobial stewardship.

In conclusion, this study showed that the cost for DPC is higher in an acute compared to an elective setting. DPC had minimal short‐term cost savings, and further research is needed to determine any long‐term health economic impact on primary care consultations, hospital attendances, antimicrobial resistance, healthcare‐associated infections, and health‐related quality of life.

## Author Contributions

R.Be. contributed to data analysis and led the write‐up. R.B. contributed to study design, obtaining, analysing, and interpreting of data, and writing up of manuscript. K.K. conducted screening, risk stratifications, and DPCs at Oxford, managed data, including validation, and inputted with the development of the economic model. B.Y.N. conducted screening, risk stratifications, and DPCs at Oxford, managed data including validation, and inputted with the development of the economic model. B.J. contributed to the obtaining, analysing, and the interpretation of data. C.M. contributed to interpretation of data and the writing up of the manuscript. C.T. contributed to the interpretation of data and the writing up of manuscript. L.S. contributed to study design and protocol, funding acquisition, the interpretation of data, and writing up of manuscript. S.A.M. contributed to study design and protocol, funding acquisition, the interpretation of data and write up of the manuscript. M.T.K. contributed to funding acquisition, study design and protocol, data interpretation and write up. R.M.M. led the study design, data analysis and the interpretation and write‐up and is the guarantor of this study.

## Conflicts of Interest

RMM is supported in part by the National Institute for Health and Care Research (NIHR) Leeds Biomedical Research Centre (BRC) (NIHR 203331). MTK has received research grants from NIHR, MRC CiC, FSA, and GCRF (University of Birmingham) for work unrelated to that presented in this manuscript. He is co‐author of BSACI penicillin allergy guidelines and previous Chair of BSACI Equality, Diversity, and Inclusion Working Group. The views expressed are those of the author(s) and not necessarily those of the NHS, the NIHR, or the Department of Health and Social Care.

## Supporting information


Data S1.

## Data Availability

The data that supports the findings of this study are available in the Supporting Information of this article.
