# Supplementary material for: An Economic Evaluation of Direct Oral Penicillin Challenge for De‐Labelling Low Risk Patients With a Penicillin Allergy Label
Source: Clin Exp Allergy. 2025 Feb 5;55(5):378–90. doi: 10.1111/cea.14633 (PMC12088836; doi:10.1111/cea.14633)
Supplement: Supplementary file 1 — Data S1. [file CEA-55-378-s001.zip › 3_CEA14633-sup-0001-Supinfo.docx]

**Supplementary file**

**C**ontents

[Inclusion criteria 2](#_Toc189493389)

[Table S1: Inclusion and exclusion criteria at screening. 2](#_Toc189493390)

[Risk stratification criteria 2](#_Toc189493391)

[Unit costs 4](#_Toc189493392)

[Table S2: Unit costs of SPACE resource inputs 4](#_Toc189493393)

[Duration of screening and risk stratification 4](#_Toc189493394)

[Table S3: Time taken to complete the data collection recorded on REDCap (minutes) 5](#_Toc189493395)

[Search strategy for review of economic studies of penicillin allergy delabelling 6](#_Toc189493396)

[Ovid MEDLINE(R) ALL <1946 to November 10, 2023> 6](#_Toc189493397)

[Details of derivations for extrapolations of costs of and effects of DPC 7](#_Toc189493398)

[Detailed results by site 8](#_Toc189493399)

[Birmingham 8](#_Toc189493400)

[Table S4: Costs of staff inputs for SPACE intervention steps at Birmingham study site (£) 8](#_Toc189493401)

[Oxford 8](#_Toc189493402)

[Table S5: Costs of staff inputs into delivering SPACE intervention steps at Oxford site (£) 8](#_Toc189493403)

[Leeds 9](#_Toc189493404)

[TableS6: Costs of staff inputs into delivering SPACE intervention steps at Leeds site (£) 9](#_Toc189493405)

[FigureS1: Staff time and costs for SPACE delivering activities by study site 10](#_Toc189493406)

[Antibiotic Medication use and costs 11](#_Toc189493407)

[TableS7: Costs of antibiotic medication use in pre-surgical patients de-labelled before surgery (£) 11](#_Toc189493408)

[TableS8: Costs of antibiotic medication use in AMU/IDU therapeutic de-labelled patients (£) 11](#_Toc189493409)

[Summary of costs up to 3 days after DPC 12](#_Toc189493410)

[Table S9: Summary of costs up to 3 days after DPC (£) 12](#_Toc189493411)

[Sensitivity analysis 13](#_Toc189493412)

[TableS10: Sensitivity Analysis: staff costs per de-labelled patient (£) 13](#_Toc189493413)

[TableS11: Sensitivity Analysis: extrapolated net total costs by patient subgroup and study site (£) 13](#_Toc189493414)

[Value of Information 13](#_Toc189493415)

[TableS12: Expected value of perfect information: modelled 5-year time horizon after DPC 13](#_Toc189493416)

# Inclusion criteria

### Table S1: Inclusion and exclusion criteria at screening.

| **Inclusion criteria:** |
| --- |
| 1. Patients with a current PAL, ≥18 years, with capacity to give informed consent |
| **Exclusion criteria:** |
| **1.** Clinically unstable patients, i.e., unstable cardio-respiratory status (eg: respiratory failure, cardiac failure, pre-hepatic encephalopathy etc.) |
| **2.** History of serious non-immediate systemic hypersensitivity reactions (HSRs) to penicillin |
| **a.** *Documented Steven Johnson syndrome (SJS), toxic epidermal necrolysis (TENS), acute exanthematous generalised pustulosis (AEGS), erythema multiforme, haemolytic anaemia, vasculitis, acute interstitial nephritis* |
| **3.** Those deemed unsuitable for medical reasons (unlikely to comply with study protocol) |
| **4.** Pregnant |
| **5.** Breast feeding |
| **6.** Concomitant COVID-19 infection |
| **7.** Those participating in any other research currently or those who have participated in research involving medicinal product, medical devices and/or other intervention in preceding 6 weeks. |
| **8.** Patients currently receiving Omalizumab or those who have received Omalizumab within 6 months prior to proposed DPC |
| **9.** Patients currently taking antihistamine and unable to temporarily withdraw for the proposed DPC |
| **10.** Patients with significant psychological/psychiatric conditions such as severe anxiety, severe depression, dementia, schizophrenia etc., that is deemed unsuitable for informed consent |

# Risk stratification criteria

**‘POST INITIAL TRIAGE’ SCREENING LOG FOR RISK STRATIFICATION**

**RISK STRATIFICATION CRITERIA: This proforma must be completed in conjunction with study protocol**

*Low Risk:* Those with one or more of the following:

- history of nonspecific symptoms *only* (eg: headache, isolated dizziness, gastrointestinal symptoms)
- Thrush *only*, no other symptoms
- mild ‘benign##’ rash (during treatment course and not ≤1hr after first dose)
- Pruritus without rash
- those with an indeterminate history but non-life threatening and did not require hospitalisation
- Remote (>10 years) reactions without features of an IgE mediated reaction
- Tolerated treatment with amoxicillin/co-amoxiclav since registration of PenA label
- No history of an ‘index episode’ but has been advised to avoid penicillins due to family history

*##benign rash: Check list for a ‘benign’ rash - should satisfy all of the following:*

- *Non-blistering, not painful, non-desquamating, non-bruising*
- *No associated mouth ulcers/genital ulcers*
- *Not systemically unwell due to the reaction*
- *Not hospitalised*

*If any of the above are not satisfied or relevant information is not available, patient will be stratified as ‘high risk’ (see below).*

*High Risk (not for DPC):* Those with any one or more of the following:

- severe, uncontrolled or brittle asthma
- severe COPD
- heart failure or severe impairment in cardiac function
- symptoms suggestive of an IgE mediated reaction or anaphylaxis after administration of penicillins
- blistering, painful, desquamating or bruising rash
- symptoms requiring hospital admission or treatment
- history of angioedema as a part of index reaction

**PROFORMA (To be completed by research nurse or research pharmacist conducting Direct oral Penicillin Challenge [DPC])**

***Note: Review information regarding suitability for this study in ‘screening log’ and proceed with risk stratification only if patient is suitable for inclusion in the study. In female patients of child bearing potential, check up to date pregnancy status via clinical history and exclude if pregnant, document accordingly in study documents and hospital records.***

# Unit costs

### Table S2: Unit costs of SPACE resource inputs

| **Item** | **Definition** | **Unit cost (£)** | **Source** |
| --- | --- | --- | --- |
| ***Training*** | | | |
| Instructor time, Senior Research pharmacist | Hourly, Band 8a | 73 | Jones and Burns [1]. Includes wages/salaries, salary on costs, management and non-care staff overheads, non-staff overheads (including drugs, travel/transport, training and conferences, supplies and services, and utilities) and land and building space. |
| Instructor time, Allergy specialist | Hourly, Senior Consultant | 123 |  |
| ***Staff time delivering SPACE*** | | | |
| Research Pharmacist | Hourly, Band 8a | 73 | Jones and Burns [1]. Includes wages/salaries, salary on costs, management and non-care staff overheads, non-staff overheads (including drugs, travel/transport, training and conferences, supplies and services, and utilities) and land and building space. |
| Senior Research Nurse | Hourly, Grade 6 | 51 |  |
| Data Manager | Hourly, Band 4 | 35 |  |
| Allergy specialist | Hourly, Senior Consultant | 123 |  |
| Consultant managing patient | Hourly, Senior Consultant | 123 |  |
| ***DPC Tests consumables*** | | | |
| Medications for prolonged DPC | 500mg oral Amoxicillin + 250mg oral Amoxicillin twice daily for three days | 0.140 | EMIT [2] |
| Medications for therapeutic DPC | 500mg oral Amoxicillin | 0.025 |  |

# Duration of screening and risk stratification

According to prospective data collected using the Research Electronic Data Capture (REDCap) secure web application for online surveys and databases hosted at Leeds Teaching Hospital Trust [3], screening of participants took an average of 6.3 (SD 3.4) minutes in Birmingham (n = 685/795, 14% missing data, Figure 2), 5.6 (SD 4.4) minutes in Oxford (n=466/472; 1.3% missing data, Figure 2) and 10.0 (SD 0) minutes in Leeds (n=3/990, 99.7% missing data, Figure 2) (Table S3,). This contrasted with the average estimates provided by retrospective staff time input questionnaires, respectively 18.3 (n=795), 20.5 (n=472) and 3.7 (n=577) minutes (Figure S1). We used the individual variation in the prospective REDCap data from Birmingham to incorporate uncertainty into our cost parameters (Table S3).

### Table S3: Time taken to complete the data collection recorded on REDCap (minutes)

| **Phase** | **Birmingham** | | **Leeds** | | **Oxford** | |
| --- | --- | --- | --- | --- | --- | --- |
|  | N | Mean (SD) | N | Mean (SD) | N | Mean (SD) |
| Screening | 685 | 6.3 (3.4) | 3 | 10.0 (0) | 466 | 5.6 (4.4) |
| Risk Stratification | 90 | 51.4 (30.7) | 14 | 14.1 (13.4) | 76 | 19.0 (5.2) |

# Search strategy for review of economic studies of penicillin allergy delabelling

This search was undertaken for a study (currently under review) of the costs of penicillin allergy labels. We copy below the latest update search strategy.

## Ovid MEDLINE(R) ALL <1946 to November 10, 2023>

Search Date: 14 November 2023

1 Economics/ 27513

2 exp Economics, Dental/ 4074

3 exp Economics, Nursing/ 4013

4 exp Economics, Medical/ 14404

5 exp Economics, pharmaceutical/ 3113

6 exp Economics, Hospital/ 25763

7 exp "Costs and Cost Analysis"/ 267176

8 exp "Fees and Charges"/ 31420

9 exp budgets/ 14157

10 exp "Value of Life"/ec [Economics] 253

11 budget*.tw. 36295

12 cost*.ti. 146158

13 (cost* adj2 (effective* or utilit* or benefit* or minimi* or evaluat* or analy* or study or studies or consequenc* or compar* or efficienc* or variable* or unit or estimate*)).ab. 239324

14 (economic* or pharmacoeconomic* or pharmaco-economic*).tw. 376662

15 (price or prices or pricing).tw. 52366

16 (financ* adj2 (cost* or data or "health care" or healthcare)).tw. 11567

17 (fee or fees).tw. 21847

18 (value adj1 (money or monetary)).tw. 885

19 quality-adjusted life years/ 15921

20 (eq-5d* or eq5d* or euroquol* or euroqol* or euroqual* or euro-quol* or euro-qol* or euro-qual*).tw. 17384

21 exp models, economic/ 16242

22 markov chains/ 16047

23 quality adjusted life.tw. 17665

24 (qaly or qalys or qald or qale or qtime).tw. 14616

25 disability adjusted life.tw. 5524

26 (daly or dalys).tw. 4851

27 Disability-Adjusted Life Years/ or Healthy Life Expectancy/ [new 2022] 275

28 "Global Burden of Disease"/ [new 2017] 2044

29 health* year* equivalent*.tw. 40

30 (hye or hyes).tw. 75

31 (hui1 or hui2 or hui3).tw. 447

32 disutil*.tw. 649

33 standard gamble*.tw. 910

34 Utility value*.tw. 2271

35 (time trade off or time tradeoff).tw. 1660

36 (hqol or h qol or hrqol or hr qol).tw. 24361

37 (pqol or qls).tw. 469

38 (sf6d or sf 6d or short form 6d or shortform 6d or sf sixd or sf six d).tw. 1013

39 exp animals/ not (exp animals/ and exp humans/) 5169565

40 exp Veterinary Medicine/ 26242

41 exp Animal Experimentation/ 10379

42 ((energy or oxygen* or metaboli*) adj3 (expenditure* or cost*)).tw. 42571

43 or/39-42 5222761

44 or/1-38 966024

45 44 not 43 901973

46 exp penicillins/ or exp beta-Lactams/ 140281

47 exp Drug Hypersensitivity/ 49878

48 Hypersensitivity/ or Anaphylaxis/ or exp Hypersensitivity, Immediate/ or exp hypersensitivity, delayed/ 362781

49 46 and (47 or 48) [MESH penicillin allergy] 6189

50 (allerg* or hypersensitiv* or intoleran* or anaphyla*).tw. 348070

51 (penicillin* or beta-lactam*).tw. 105253

52 (amoxicillin or Amdinocillin or Cyclacillin or Methicillin or Nafcillin or Cloxacillin or Floxacillin or Dicloxacillin or Penicillanic Acid or Tazobactam or Ampicillin or Carbenicillin or Carfecillin or Sulbenicillin or Sulbactam or Ticarcillin).tw. [named penicillins] 93572

53 50 and (51 or 52) [penicillin allergy] 6006

54 49 or 53 9356

55 45 and 54 [Ec Eval penicillin allergy - all] 218

56 (delabel* or de-label* or mislabel* or Mis-label*).tw,kf. 1925

57 ((antibiotic* or antimicrobial*) adj stewardship).tw,kf. 10492

58 Antimicrobial Stewardship/ 3451

59 or/56-58 [delabelling terms] 12933

60 47 or 48 or 50 [hypersensitivity terms] 578114

61 59 and 60 [delabelling allergies] 582

62 45 and 61 [Ec eval - delabelling] 53

63 55 or 62 [Ec eval delabelling or penicillin allergies] 226

# Details of derivations for extrapolations of costs of and effects of DPC

Souza-Pinto et al used a simple algebraic model populated with data identified from a review of the health service research literature [4]. They reported a net monetary benefit (i.e., cost savings) per ‘low risk’ patient of £4813 (at year 2020 exchange rates) over 5 years [4]. We could not identify any new evidence on the number of avoided subsequent GP contacts per patient per year by removing PAL. Therefore, we used the same estimate as Souza-Pinto et al evaluated at UK unit costs with a 3.5% annual discounting rate as recommended by NICE guidelines [1,4,5]. The same applied to the 12-week readmission relative risk ratio, which was derived from a prospective matched cohort of all patients admitted to a Dutch hospital between 2013 and 2014 [6]. Since ONS data routinely report hospital readmission rates up to 30 days, we used the 4- to 12-week readmission rate ratio reported in the study by Van Dijk et al. to convert the ONS readmission rates to 90 days [6,7].

The consequences of de-labelling would vary depending on the clinical setting. Patients in AMU/IDU undergoing 'therapeutic de-labelling' and who were not already on a first-line penicillin-containing antibiotic would have immediate clinical benefit. For example, de-labelling these patients permits them to be switched from less preferable second-line therapy to the first-line penicillin-containing treatment. Similarly, for patients in the pre-surgical group, if the first-line prophylaxis contained penicillin, and they were de-labelled pre-surgery, they would now be able to receive this instead of a second-line antibiotic.

Haematology-Oncology patients did not realize the immediate benefit from a successful DPC. However, many of these patients are immunocompromised and at increased risk of infection and neutropenic sepsis (NS). A successful DPC would enable them to receive penicillin treatment for future NS events. We have conducted but not reported the results of hypothetical exploratory scenarios of these effects over 12 months after delabelling, which resulted in small medication savings, these are available from the authors.

# Detailed results by site

## Birmingham

### Table S4: Costs of staff inputs for SPACE intervention steps at Birmingham study site (£)

|  | **Research pharmacist** | **Study consultant** | **Research nurse** | **Admin/data manager** | **Allergy specialist** | **Total** |
| --- | --- | --- | --- | --- | --- | --- |
| **Training*** | 5037 | 1476 | 408 | 873 |  | **7794** |
| **Screening** | 7858 |  | 5490 | 933 |  | **14281** |
| **Consent** | 5329 |  | 3097 |  |  | **8426** |
| **Risk stratification (RS)** | 17551 | 1927 | 34 |  | 62 | **19574** |
| **Communication of RS outcome via telephone** | 1034 |  |  |  |  | **1034** |
| **DPC** | 7531 | 5997 |  |  |  | **13528** |
| **Day 5 follow-up** | 1144 |  |  |  |  | **1144** |
| **De-labelling and AE/SAE** | 1703 | 461 |  |  | 318 | **2482** |
| Total | 47187 | 9861 | 9029 | 1806 | 380 | **68263** |
| *Training costs refer to the staff time of i) attending training and ii) instructors delivering training  DPC: Direct Penicillin Challenge. AE/SAE: Adverse Event/Severe Adverse Event | | | | | | |

## Oxford

### Table S5: Costs of staff inputs into delivering SPACE intervention steps at Oxford site (£)

|  | **Research pharmacist** | **Study consultant** | **Research nurse** | **Admin/data manager** | **Allergy specialist** | **Total** |
| --- | --- | --- | --- | --- | --- | --- |
| **Training*** | 1460 | 1476 |  |  | 408 | **3344** |
| **Screening** | 4434 |  |  |  |  | **4434** |
| **Consent** | 3279 |  |  |  |  | **3279** |
| **Risk stratification (RS)** | 11344 | 1650 |  |  |  | **12994** |
| **Communication of RS outcome via telephone** | 468 |  |  |  |  | **468** |
| **DPC** | 7623 |  |  |  |  | **7623** |
| **Day 5 follow-up** | 858 |  |  |  |  | **858** |
| **De-labelling and AE/SAE** | 3103 |  |  |  | 462 | **3565** |
| **Total** | 32569 | 3126 | 0 | 0 | 870 | **36565** |
| *Training costs refer to the staff time of i) attending training and ii) instructors delivering training  DPC: Direct Penicillin Challenge. AE/SAE: Adverse Event/Severe Adverse Event | | | | | | |

## Leeds

### TableS6: Costs of staff inputs into delivering SPACE intervention steps at Leeds site (£)

|  | **Research pharmacist** | **Study consultant** | **Research nurse** | **Admin/data manager** | **Allergy specialist** | **Total** |
| --- | --- | --- | --- | --- | --- | --- |
| **Training*** |  | 1476 | 1932 | 234 |  | **3642** |
| **Screening** |  |  | 6909 | 1038 |  | **7947** |
| **Consent** |  |  | 4076 | 444 |  | **4521** |
| **Risk stratification** |  | 729 | 3241 | 173 | 513 | **4656** |
| **Communication of RS outcome via telephone** |  |  | 69 | 14 |  | **83** |
| **DPC** |  | 248 | 2422 | 474 |  | **3144** |
| **Day 5 follow-up** |  | 0 | 221 | 44 |  | **266** |
| **De-labelling and AE/SAE** |  | 0 | 430 | 3 | 0 | **433** |
| **Total** | 0 | 2453 | 19301 | 2425 | 513 | **24691** |
| *Training costs refer to the staff time of i) attending training and ii) instructors delivering training  DPC: Direct Penicillin Challenge. AE/SAE: Adverse Event/Severe Adverse Event | | | | | | |

##

## FigureS1: Staff time and costs for SPACE delivering activities by study site


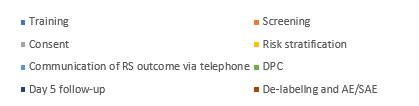


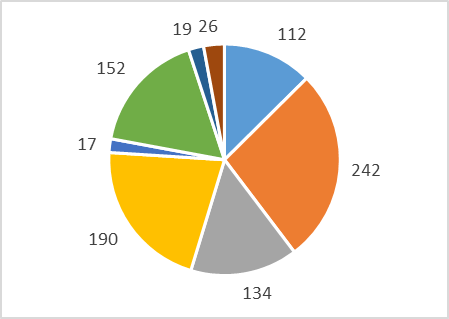

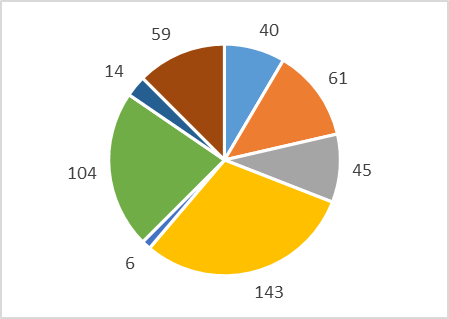

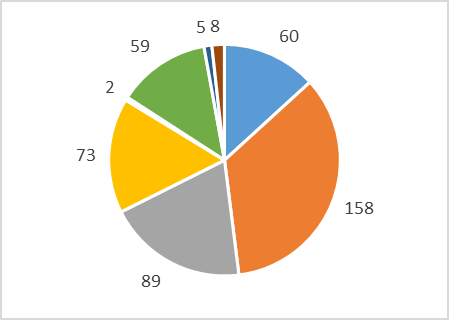

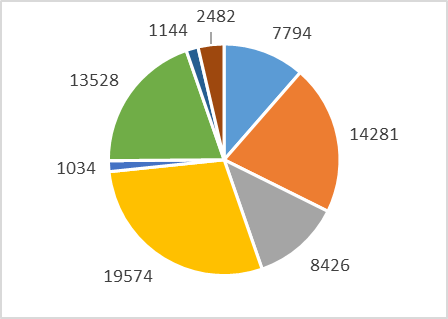

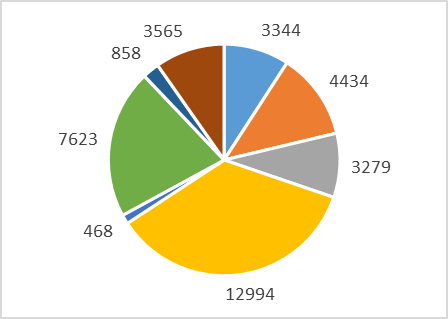

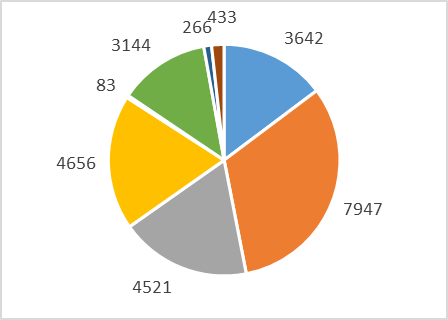


Notes: DPC: Direct Penicillin Challenge. AE/SAE: Adverse Event/Severe Adverse Event

**Leeds**

**Oxford**

**Birmingham**

**Cost (£)**

**Time (minutes)**

# Antibiotic Medication use and costs

### TableS7: Costs of antibiotic medication use in pre-surgical patients de-labelled before surgery (£)

| **Study Site** | **Specialty** | **Number of patients** | **Antibiotic cost (no PAL)** | **Antibiotic cost (PAL)** | **Cost difference** |
| --- | --- | --- | --- | --- | --- |
| Birmingham | Gastro-Intestinal | 1 | 0.84 | 1.69 | -0.85 |
| Birmingham | Urology | 1 | 2.16 | 10.59 | -8.43 |
| Birmingham | (ENT (n=2)), bariatric surgery (n=2), urology | 5 | 0.00 | 0.00 | 0.00 |
| Leeds | Gynaecology | 1 | 2.92 | 2.92 | 0.00 |
| Leeds | Colorectal/Gastro-Intestinal | 4 | 1.27 | 9.05 | -7.78 |
| Leeds | Urology | 3 | 1.21 | 1.21 | 0.00 |
| Leeds | Breast | 1 | 0.84 | 14.58 | -13.74 |
| Leeds | General | 4 | 0.00 | 1.29 | -1.29 |
| Oxford | ENT | 1 | 1.79 | 1.81 | -0.02 |
| Oxford | Urology | 1 | 1.81 | 3.85 | -2.04 |
| Oxford | Maxillofacial, Interventional Radiology, ENT | 3 | 3.85 | 0.00 | 3.85 |
| Oxford | Maxillofacial surgery | 1 | 0.84 | 1.69 | -0.85 |
| Total | Weighted mean | 26 | 1.21 | 3.16 | -£1.95 (95% CI: -3.68, -0.22) |
| Notes: PAL: Penicillin Allergy Label. ENT: Ear, Nose, Throat | | | | | |

### TableS8: Costs of antibiotic medication use in AMU/IDU therapeutic de-labelled patients (£)

| **Patient** | **Actual route** **after de-labelling** | **Cost of antibiotics after penicillin allergy label removed**  **(a)** | **Alternative antibiotic route (if patient had retained PAL)** | **Cost of alternative antibiotics (if patient had retained PAL) (b)** | **Cost difference**  **(a-b)** |
| --- | --- | --- | --- | --- | --- |
| 1 | Amoxicillin 500mg PO three times a day | 0.50 | Doxycycline 200mg, once daily PO, for one day, then 100mg PO once daily for four days | 0.23 | 0.27 |
| 2 | Amoxicillin 500mg PO three times a day | 0.50 | Doxycycline 200mg PO once daily for one day, then 100mg PO once daily for four days | 0.23 | 0.27 |
| 3 | Amoxicillin 250mg twice daily for one day PO, then piperacillin/tazobactam 4.5g IV two times a day for four days | 12.79 | Ciprofloxacin 400mg IV twice daily | 25.45 | -12.66 |
| 4 | Amoxicillin 1g three times a day PO | 1.00 | Ciprofloxacin 500mg PO twice daily | 0.55 | 0.45 |
| 5 | Co-amoxiclav 1.2g IV three times a day for three days, then piperacillin/tazobactam 4.5g IV twice a day for 2 days | 11.41 | Ceftriaxone 2g IV once daily and clarithromycin 500mg PO twice daily | 7.74 | 3.67 |
| 6 | Piperacillin/tazobactam 4.5g IV three times a day | 23.93 | Ceftazidime 2g IV three times a day | 24.90 | -0.975 |
| Mean | | 8.20 |  | 9.85 | -1.49  (95% CI: -7.46, 4.47) |
| Note: Duration costed is 5 days. Medication costs are from eMIT National Database [2]  PO: oral. IV: intravenous. PAL: Penicillin Allergy Label | | | | | |

### Summary of costs up to 3 days after DPC

### Table S9: Summary of costs up to 3 days after DPC (£)

|  | **Costs per screened patient** | **Costs per patient undergoing oral challenge** | **Cost per de-labelled patient** |
| --- | --- | --- | --- |
| **Staff time delivering DPC** | 50.60 | 906.87 | 936.61 |
| **Consumables (including DPC antibiotics)** | 0.01 | 0.10 | 0.11 |
| **Antibiotic use in presurgical patients^a^** | -0.02 | -0.43 | -0.44 |
| **Antibiotic use in AMU/IDU patients^b^** | -0.00^c^ | -0.07 | -0.07 |
| **Total** | 50.59 | 906.47 | 936.21 |
| ^a^ By presurgical patients de-labelled before surgery; ^b^ For therapeutically de-labelled patients; Value is negative but presented as zero due to rounding. | | | |

# Sensitivity analysis

### TableS10: Sensitivity Analysis: staff costs per de-labelled patient (£)

|  | **Birmingham** | **Oxford** | **Leeds** |
| --- | --- | --- | --- |
| Base Case | 1344 | 722 | 679 |
| With training costs | 1516 | 795 | 796 |
| Without screening costs | 1026 | 626 | 423 |
| Without screening and day 5 follow-up | 1001 | 607 | 414 |

### TableS11: Sensitivity Analysis: extrapolated net total costs by patient subgroup and study site (£)

|  | **Birmingham** | **Oxford** | **Leeds** |
| --- | --- | --- | --- |
| Base Case | 719 | 88 | 52 |
| With training costs | 892 | 160 | 170 |
| Without screening costs | 402 | -9 | -204 |
| Without screening and day 5 follow-up | 376 | -27 | -213 |

# Value of Information

The value of conducting further research would be driven by measuring the costs of GP visits over a 4.5-year period. For the average patient over this period, avoiding the extra 13 GP visits expected per patient with a PAL, relative to without a PAL, would amount to a present discounted (at annual 3.5% rate) value of cost savings of £603 (including prescription costs), as opposed to £45 savings from 90-day hospital readmissions (including the reduction in length of hospital stay).

### TableS12: Expected value of perfect information: modelled 5-year time horizon after DPC

| **Parameter** | **Expected Value of perfect partial information per patient (£)** | | |
| --- | --- | --- | --- |
|  | Birmingham model | Oxford Model | Leeds model |
| **AMU/IDU** | 0 | 12.68 | 0.03 |
| **Pre-surgical** | 0 | 9.69 | 29.14 |
| **Haematology-Oncology** | 0 | 11.80 | 99.73 |

**References**

1. Jones KC, Burns A. Unit costs of health and social care PSSRU; 2021.
2. Department of Health and Social care. Drugs and pharameutical electronic market information tool (eMIT). Update 22 March 2023. [Accessed June 2023] <https://www.gov.uk/government/publications/drugs-and-pharmaceutical-electronic-market-information-emit>
3. PA Harris, R Taylor, R Thielke, J Payne, N Gonzalez, JG. Conde, Research electronic data capture (REDCap) – A metadata-driven methodology and workflow process for providing translational research informatics support, J Biomed Inform. 2009 Apr;42(2):377-81.
4. Sousa-Pinto B, Blumenthal KG, Macy E, et al. Penicillin Allergy Testing Is Cost-Saving: An Economic Evaluation Study. *Clin Infect Dis* 2021; **72**(6): 924-38.
5. NICE health technology evaluations: the manual; Process and methods.4 Economic Evaluation. Published: 31 January 2022. NICE. Accessed June 2023. <https://www.nice.org.uk/process/pmg36/chapter/economic-evaluation>
6. van Dijk SM, Gardarsdottir H, Wassenberg MW, Oosterheert JJ, de Groot MC, Rockmann H. The High Impact of Penicillin Allergy Registration in Hospitalized Patients. *J Allergy Clin Immunol Pract* 2016; **4**(5): 926-31.
7. NHS Digital. Compendium – Emergency readmissions to hospital within 30 days of discharge. October 2022. [Accessed July 2023].
8. UK Health Security Agency. English surveillance programme for antimicrobial utilisation and resistance (ESPAUR) Report 2021 to 2022. London: UK Health Security Agency; 2022.
